# Real‐World Validation of the Clinical Distinctiveness of IDH‐Mutant Glioblastoma in the SEER Transition Era: A Population‐Based Study Integrating Machine Learning

**DOI:** 10.1002/brb3.71641

**Published:** 2026-08-11

**Authors:** Dewei Du, Amu Jike, Dongnan Yu, Shizheng Tan, Shuming Wang, Shun Li

**Affiliations:** ^1^ Department of Neurosurgery Affiliated Hospital of North Sichuan Medical College Nanchong China; ^2^ Department of Neurosurgery Liangping People's Hospital of Chongqing Chongqing China

**Keywords:** glioblastoma, IDH mutations, machine learning, Random Forest, SEER database, survival analysis

## Abstract

**Introduction:**

The 2021 WHO classification reclassified “IDH‐mutant glioblastoma (GBM)” as “Astrocytoma, IDH‐mutant, grade 4.” This study aims to provide real‐world validation of this reclassification using the specific ICD‐O‐3 code (9445/3) from the Surveillance, Epidemiology, and End Results (SEER) “Transition Era” (2018–2022) and develop a machine learning (ML)‐based prognostic model.

**Methods:**

Patients diagnosed with IDH‐mutant GBM (9445/3) and GBM NOS (9440/3) were identified. Propensity Score Matching (PSM) and Inverse Probability of Treatment Weighting (IPTW) were employed to minimize bias. A doubly robust Cox regression model was constructed to quantify survival benefits. Nine ML algorithms were integrated to develop a prognostic signature, which was interpreted using SHAP (Shapley Additive exPlanations) analysis.

**Results:**

Of 13,443 patients, 312 were IDH‐mutant. After matching, the IDH‐mutant group exhibited significantly superior overall survival (OS) and cancer‐specific survival (CSS) (*p* < 0.001). IDH mutation emerged as a potent independent favorable prognostic factor, associated with a 65.0% lower mortality risk (HR = 0.350, *p* < 0.001). Subgroup analysis confirmed robust benefits from chemotherapy. The Random Forest (RF) model achieved the best performance (Test AUC = 0.698). SHAP analysis identified IDH status, chemotherapy, and age as the top predictors.

**Conclusion:**

This study provides compelling evidence in support of the clinical rationale for the WHO 2021 reclassification. Despite a favorable prognosis, aggressive multimodal therapy was strongly associated with improved survival, though potential indication bias necessitates cautious interpretation and prospective validation. The developed ML model serves as a robust tool for personalized risk stratification.

## 1 Introduction

Glioblastoma (GBM) represents the most common and lethal primary malignant brain tumor in adults, historically characterized by high heterogeneity and dismal clinical prognosis (Price et al. 2024). Despite standard multimodal therapy, including maximal safe resection, radiotherapy, and adjuvant temozolomide, the median overall survival remains less than 15 months, with a 5‐year survival rate below 7% (Stupp et al. 2005; Stupp et al. 2009). For decades, the diagnosis of GBM relied primarily on histological hallmarks, resulting in a biologically disparate patient population under a single diagnostic label (Ceccarelli et al. 2016). The discovery of isocitrate dehydrogenase (IDH) mutations in 2008 marked a paradigm shift in neuro‐oncology (Parsons et al. 2008). Studies have confirmed that this mutation remodels the epigenetic landscape by producing the oncometabolite 2‐hydroxyglutarate (2‐HG), conferring distinct pathogenesis and a significantly superior survival prognosis compared to wild‐type tumors (Dang et al. 2009; Turcan et al. 2012; Yan et al. 2009).

Given these fundamental biological differences, the World Health Organization (WHO) Classification of Tumors of the Central Nervous System has undergone decisive evolution. Although the 2016 classification first introduced the concept of “GBM, IDH‐mutant” (Louis et al. 2016), the 2021 WHO Classification formally removed this entity from the GBM category, reclassifying it as “Astrocytoma, IDH‐mutant, grade 4,” and reserving the term “GBM” exclusively for IDH‐wild‐type tumors (Louis et al. 2021; Eckel‐Passow et al. 2015). This nosological restructuring aims to homogenize clinical trial populations and guide precise prognostication. However, updates to real‐world cancer registry data often lag. The Surveillance, Epidemiology, and End Results (SEER) database introduced the specific ICD‐O‐3 code 9445/3 (GBM, IDH‐mutant) in 2018, creating a unique “Transition Era” (2018–2022) dataset.

Current population‐based studies have primarily relied on older codes or mixed cohorts, lacking large‐scale validation using the specific 9445/3 code to verify the clinical distinctiveness of this population relative to contemporary GBM, Not Otherwise Specified (NOS, 9440/3) (Iorgulescu et al. 2022). Although the general prognostic advantage of IDH mutations is recognized, the specific magnitude of this benefit in a matched real‐world setting and whether this subgroup exhibits differential sensitivity to standard chemotherapy remain to be fully quantified. Furthermore, traditional Cox regression models struggle to capture complex nonlinear interactions. With the growth of multi‐omics data, there is an urgent need to introduce advanced machine learning (ML) algorithms to develop precise prognostic tools superior to traditional statistical methods (Bi et al. 2019; Kickingereder et al. 2019).

Therefore, this study aims to provide comprehensive real‐world validation for the clinical rationale behind the WHO 2021 reclassification. Utilizing data from the SEER transition era (2018–2022), we employed Propensity Score Matching (PSM) to rigorously quantify the survival advantage and therapeutic sensitivity of patients coded as 9445/3. Beyond epidemiological validation, we further integrated advanced ML algorithms to develop and validate a high‐performance prognostic signature, providing clinicians with a robust tool for personalized risk stratification in the molecular era.

## 2 Materials and Methods

### 2.1 Data Source and Study Population

This retrospective cohort study utilized data from the Surveillance, Epidemiology, and End Results (SEER) database of the National Cancer Institute (NCI). Given that the specific ICD‐O‐3 histology code 9445/3 (GBM, IDH‐mutant) was formally introduced in 2018, the study period was restricted to 2018 to 2022, defined as the “Transition Era” of the coding system.

We identified patients with a histological diagnosis of “GBM, IDH‐mutant” (ICD‐O‐3 code 9445/3) and “GBM, NOS” (ICD‐O‐3 code 9440/3). Inclusion criteria were: (1) histologically confirmed diagnosis; (2) age ≥ 18 years at diagnosis; and (3) complete follow‐up information with known survival time. A complete‐case analysis approach was utilized; therefore, exclusion criteria included diagnoses based solely on autopsy or death certificate, and missing data for essential demographic or treatment variables.

### 2.2 Variable Definitions and Outcomes

Covariates extracted for analysis were selected based on clinical relevance and established neuro‐oncological prognostic factors, which included: (1) demographic characteristics: age, sex, race, marital status, and median household income; (2) tumor characteristics: primary site, laterality, tumor size, and SEER Combined Summary Stage; and (3) treatment factors: surgery, radiotherapy, chemotherapy, and systemic therapy.

The primary endpoint was overall survival (OS), defined as the time from diagnosis to death from any cause. The secondary endpoint was cancer‐specific survival (CSS), defined as the time from diagnosis to death explicitly attributed to the brain tumor.

### 2.3 PSM and Inverse Probability of Treatment Weighting (IPTW)

To minimize selection bias and balance baseline characteristics between the IDH‐mutant (9445/3) and NOS (9440/3) cohorts, two complementary statistical techniques were employed: (1) PSM: a multivariable logistic regression model was constructed to calculate propensity scores, incorporating all baseline covariates (age, sex, race, tumor characteristics, and treatment modalities) (we performed 1:4 nearest neighbor matching with a caliper of 0.02 to ensure matching precision) and (2) IPTW: to retain complete sample information and enhance generalizability, stabilized weights were calculated based on propensity scores to construct a Weighted Pseudo‐population.

Covariate balance was assessed using the Standardized Mean Difference (SMD), with an SMD < 0.10 considered indicative of negligible group differences.

### 2.4 Statistical Analysis

Baseline characteristics were compared using the chi‐square test (categorical variables) or the Mann–Whitney U test (continuous variables). Survival curves were generated using the Kaplan–Meier method, and differences were evaluated via the Log‐rank test.

Before survival modeling, the proportional hazards (PH) assumption was explicitly verified for all covariates using Schoenfeld residuals, and no significant violations were observed. Furthermore, potential influential observations were assessed using deviance residuals and dfbeta values to ensure model stability (**Figure** **S1**).

To rigorously quantify the survival benefit of the IDH‐mutant subtype, three hierarchical Cox proportional hazards models were constructed: (1) Model 1 (Unadjusted): a standard multivariable Cox regression in the original, unmatched cohort, adjusting for all clinical covariates; (2) Model 2 (PSM Adjusted): a univariable Cox regression applied to the PSM‐matched cohort, evaluating only the group variable; (3) Model 3 (Doubly Robust): a multivariable Cox regression performed *within the PSM‐matched cohort*, simultaneously adjusting for the group variable and all clinical covariates. In this doubly robust approach, the propensity score was used exclusively for the initial matching step and was not included as an additional covariate. To appropriately account for the non‐independence of observations within the matched pairs and correct the variance estimation, robust standard errors (sandwich estimators) were utilized in this final model. Model 3 served as our primary analysis.

Based on Model 3, subgroup analyses were performed by demographic and treatment characteristics to assess the consistency of survival benefits and explore potential interactions. All statistical analyses (including ML) were conducted using Python (version 3.10.3) and R software (version 4.4.1). A two‐sided *p* value < 0.05 was considered statistically significant.

### 2.5 ML‐Based Prognostic Model Construction

To develop a precise prognostic tool for GBM patients, we integrated nine mainstream ML algorithms: Gradient Boosting Decision Tree (GBDT), K‐Nearest Neighbors (KNN), Linear Discriminant Analysis (LDA), LightGBM, Random Forest (RF), Stacking Ensemble, Support Vector Machine (SVM), Artificial Neural Network (ANN), and XGBoost (Kourou et al. 2015).

The total cohort was randomly split into a training set and a testing set at a 7:3 ratio. To prevent data leakage during model selection, hyperparameter tuning was performed using a fivefold grid search cross‐validation strictly nested within the training set. The specific search grids and optimal hyperparameter settings for all nine ML algorithms are detailed in Table S1. Model performance was comprehensively evaluated using the Area Under the Receiver Operating Characteristic Curve (AUC), sensitivity, specificity, accuracy, and *F*1‐score. The model demonstrating optimal performance in the testing set was selected as the final prognostic model. Furthermore, feature importance analysis was conducted to elucidate the contribution of each variable to model predictions.

## 3 Results

### 3.1 Baseline Characteristics and Evaluation of PSM

**FIGURE 1:**
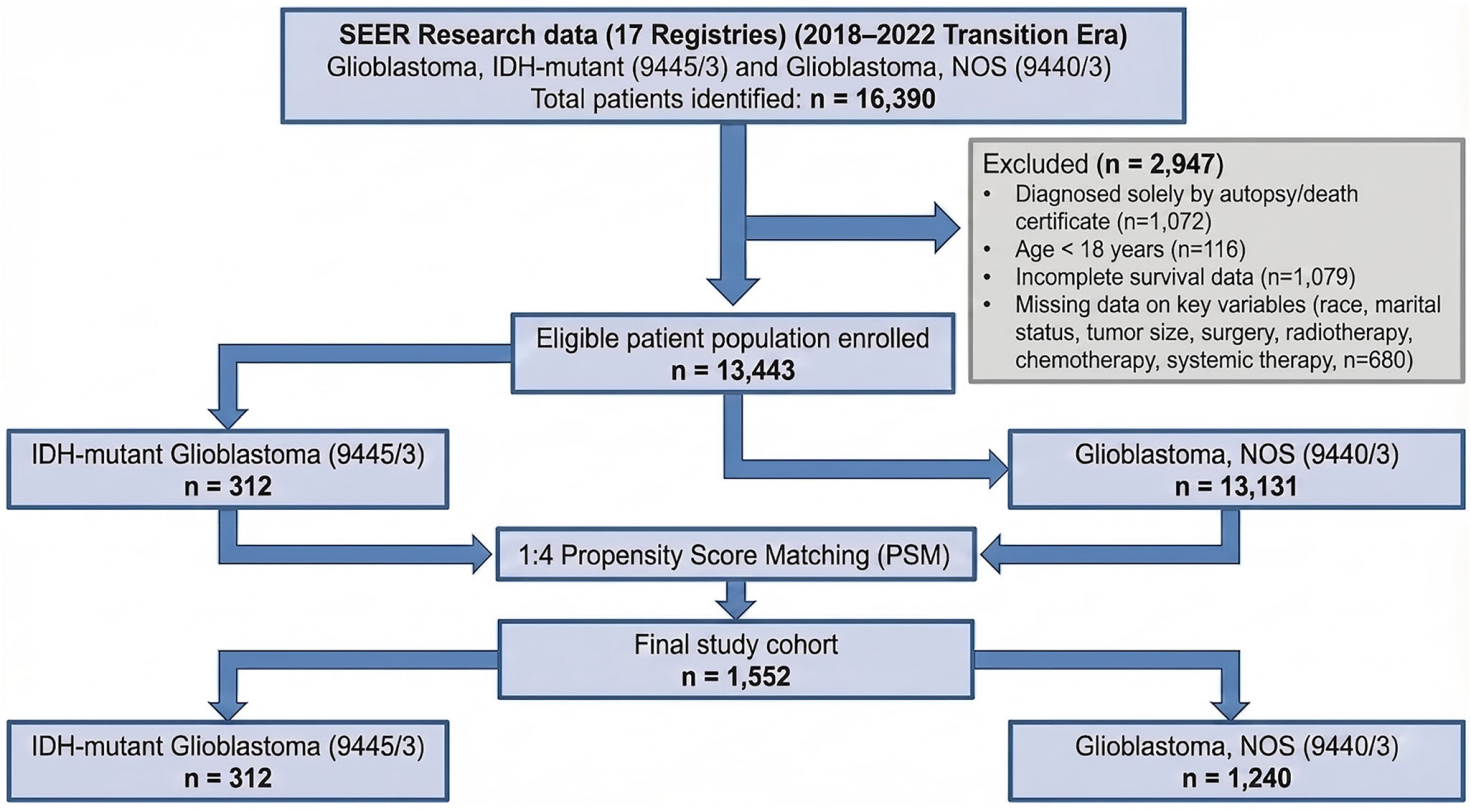
Flowchart of patient selection.

A total of 13,443 patients with GBM meeting the inclusion criteria were enrolled. Of these, 13,131 (97.7%) were diagnosed with GBM NOS, and 312 (2.3%) were identified as IDH‐mutant GBM (**Figure** **1**).

In the original unmatched cohort, significant disparities in baseline clinical characteristics were observed between the two groups (**Table** **1**). The most prominent difference was in age distribution: the IDH‐mutant group showed a distinct trend toward younger age, with 84.6% of patients aged ≤ 65 years compared to only 50.6% in the NOS group, indicating a substantial imbalance (SMD = 0.781, *p* < 0.001). Additionally, significant differences were noted in marital status (*p* < 0.001, SMD = 0.329), tumor size (*p* < 0.001, SMD = 0.270), and the receipt of systemic therapy (*p* < 0.001, SMD = 0.347). Such heterogeneity underscores the potential for severe confounding bias in direct prognostic comparisons.

**TABLE 1:** Baseline clinical characteristics of the study population before and after PSM.

Characteristic	Unadjusted		*p*‐value	SMD	PSM Adjusted		*p*‐value	SMD
	GBM NOS *N* = 13,131	GBM IDH‐mutant *N* = 312			GBM NOS *N* = 1,240	GBM IDH‐mutant *N* = 312		
Race			0.260	0.089			0.787	0.044
White	11,467 (87.3%)	263 (84.3%)			1,026 (82.7%)	263 (84.3%)		
Black	792 (6.0%)	22 (7.0%)			100 (8.1%)	22 (7.0%)		
Others	872 (6.7%)	27 (8.7%)			114 (9.2%)	27 (8.7%)		
Age			< 0.001	0.781			0.736	0.027
≤ 65	6,638 (50.6%)	264 (84.6%)			1,037 (83.6%)	264 (84.6%)		
> 65	6,493 (49.4%)	48 (15.4%)			203 (16.4%)	48 (15.4%)		
Marital status			< 0.001	0.329			0.852	0.036
Single	2,197 (16.7%)	92 (29.5%)			382 (30.8%)	92 (29.5%)		
Married	8,724 (66.4%)	187 (59.9%)			737 (59.4%)	187 (59.9%)		
Others	2,210 (16.9%)	33 (10.6%)			121 (9.8%)	33 (10.6%)		
Sex			0.071	0.108			0.666	0.032
Female	5,446 (41.5%)	113 (36.2%)			468 (37.7%)	113 (36.2%)	^†^	
Male	7,685 (58.5%)	199 (63.8%)			772 (62.3%)	199 (63.8%)		
Median household income ($)			0.638	0.030			1.000	0.004
< 100,000	9,024 (68.7%)	210 (67.3%)			837 (67.5%)	210 (67.3%)		
≥ 100,000	4,107 (31.3%)	102 (32.7%)			403 (32.5%)	102 (32.7%)		
Primary site			0.450	0.122			0.876	0.068
Supratentorial lobes	10,587 (80.6%)	261 (83.7%)			1,063 (85.8%)	261 (83.7%)		
Cerebellum and brain stem	136 (1.0%)	3 (1.0%)			8 (0.6%)	3 (1.0%)		
Ventricle	46 (0.4%)	1 (0.3%)			5 (0.4%)	1 (0.3%)		
Overlapping lesion of brain	1511 (11.5%)	35 (11.2%)			119 (9.6%)	35 (11.2%)		
Brain, NOS	851 (6.5%)	12 (3.8%)			45 (3.6%)	12 (3.8%)		
Laterality			0.211	0.105			0.751	0.048
Left	5275 (40.2%)	129 (41.3%)			541 (43.6%)	129 (41.3%)		
Right	5502 (41.9%)	139 (44.6%)			536 (43.2%)	139 (44.6%)		
Others	2354 (17.9%)	44 (14.1%)			163 (13.2%)	44 (14.1%)		
Combined summary stage			0.542	0.100			0.828	0.067
Localized	10,664 (81.2%)	253 (81.1%)			1001 (80.7%)	253 (81.1%)		
Regional	2071 (15.8%)	53 (17.0%)			207 (16.7%)	53 (17.0%)		
Distant	144 (1.1%)	1 (0.3%)	0.152	0.256	10 (0.8%)	1 (0.3%)		
Others	252 (1.9%)	5 (1.6%)			22 (1.8%)	5 (1.6%)		
Tumor size (cm)			< 0.001	0.270			0.665	0.056
< 4.5	5935 (45.2%)	102 (32.7%)			419 (33.8%)	102 (32.7%)		
≥ 4.5	5654 (43.1%)	156 (50.0%)			632 (51.0%)	156 (50.0%)		
Unknown	1542 (11.7%)	54 (17.3%)			189 (15.2%)	54 (17.3%)		
Surgery			0.001	0.244			0.777	0.045
No	2083 (15.9%)	25 (8.0%)			87 (7.0%)	25 (8.0%)		
Local/sublocal resection	10,753 (81.9%)	279 (89.4%)			1116 (90.0%)	279 (89.4%)		
Gross total resection	295 (2.2%)	8 (2.6%)			37 (3.0%)	8 (2.6%)		
Radiotherapy			0.504	0.043			0.290	0.071
No/unknown	2881 (21.9%)	63 (20.2%)			216 (17.4%)	63 (20.2%)		
Yes	10,250 (78.1%)	249 (79.8%)			1024 (82.6%)	249 (79.8%)		
Chemotherapy			0.001	0.217			0.259	0.075
No/unknown	3345 (25.5%)	52 (16.7%)			173 (14.0%)	52 (16.7%)		
Yes	9786 (74.5%)	260 (83.3%)			1067 (86.0%)	260 (83.3%)		
Systemic			< 0.001	0.347			0.286	0.072
No/unknown	4415 (33.6%)	58 (18.6%)			197 (15.9%)	58 (18.6%)		
Yes	8716 (66.4%)	254 (81.4%)			1043 (84.1%)	254 (81.4%)		

To mitigate confounding, 1:4 PSM was performed. The post‐match cohort comprised 1552 patients (312 IDH‐mutant and 1240 GBM NOS). Following matching, excellent statistical balance was achieved across all covariates (**Table** **1**, **Figure** **S2**). The SMDs for all covariates were significantly reduced to below 0.10. Furthermore, the weighted pseudo‐population constructed using IPTW also demonstrated high inter‐group balance (**Table** **S2**), with all SMDs remaining below 0.10, reinforcing the robustness of our adjustment strategy.

### 3.2 Real‐World Validation of Survival Benefits Based on Who 2021 Reclassification

**FIGURE 2:**
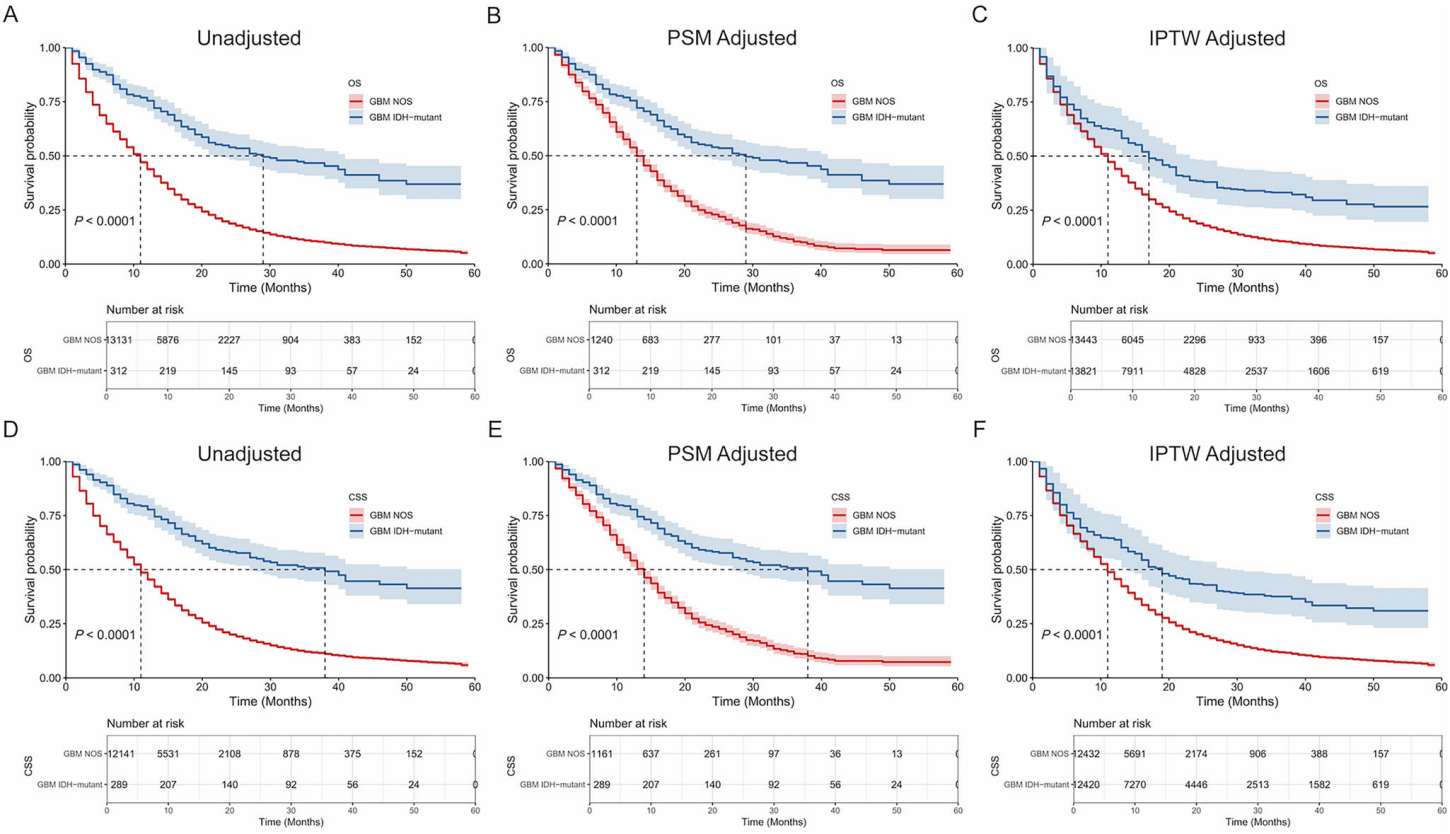
Kaplan–Meier survival analysis comparing IDH‐mutant and NOS GBM. (A, D) OS and CSS in the original unmatched cohort. (B, E) OS and CSS in the PSM cohort. (C, F) OS and CSS in the IPTW cohort. Abbreviations: OS, overall survival; CSS, cancer‐specific survival.

In the original unmatched cohort, patients with IDH‐mutant GBM exhibited significantly prolonged OS (**Figure** **2A**, *p* < 0.001) and CSS (**Figure** **2D**, *p* < 0.001) compared to the GBM NOS group.

To control for confounders, survival outcomes were further validated in the PSM‐matched cohort. Results showed that the survival curves remained significantly separated, with the IDH‐mutant group demonstrating superior OS (**Figure** **2B**, *p* < 0.001) and CSS (**Figure** **2E**, *p* < 0.001). Additionally, survival analysis based on IPTW weighting confirmed the robustness of this conclusion. In the weighted pseudo‐population, the IDH‐mutant subtype continued to show significant advantages in OS (**Figure** **2C**) and CSS (**Figure** **2F**) (*p* < 0.001).

### 3.3 Quantification of Survival Benefit and Independent Prognostic Analysis

To precisely quantify the prognostic value of IDH mutations, a doubly robust Cox regression model (Model 3) was constructed (**Table** **2**). The analysis confirmed that an IDH mutation is a potent, independent, favorable prognostic factor: compared with the NOS group, patients in the IDH‐mutant group had a 65.0% lower risk of mortality (HR = 0.350, 95% CI: 0.293–0.418, *p* < 0.001). This finding remained highly consistent across the original cohort (Model 1, HR = 0.417, 95% CI: 0.354–0.491, *p* < 0.001) and the post‐match univariable cohort (Model 2, HR = 0.397, 95% CI: 0.333–0.473, *p* < 0.001).

**TABLE 2:** Quantification of the survival benefit of IDH mutation status in glioblastoma patients based on hierarchical Cox regression models.

Variables	GBM NOS	GBM IDH‐mutant	*p* for trend
	HR (95% CI)	HR (95% CI)	
Model 1	Ref	0.417 (0.354–0.491)	< 0.001
Model 2	Ref	0.397 (0.333–0.473)	< 0.001
Model 3	Ref	0.350 (0.293–0.418)	< 0.001

Model 1 (Unadjusted): group, race, age, marital status, sex, median household income ($), primary site, laterality, combined summary stage, tumor size (cm), surgery, radiotherapy, chemotherapy, systemic.

Model 2 (PSM Adjusted): only group.

Model 3 (Doubly Robust): group, race, age, marital status, sex, median household income ($), primary site, laterality, combined summary stage, tumor size (cm), surgery, radiotherapy, chemotherapy, systemic.

Multivariable analysis concurrently identified other independent prognostic determinants (**Table** **S3**). Advanced age and combined summary stage significantly predicted poor prognosis: age >65 years (HR = 2.159, 95% CI: 1.828–2.551, *p* < 0.001) and regional stage (HR = 1.425, 95% CI: 1.212–1.676, *p* < 0.001) were associated with increased mortality risk. Conversely, aggressive multimodal therapy significantly improved survival: gross total resection (GTR) (HR = 0.594, 95% CI: 0.368–0.959, *p* = 0.033), radiotherapy (HR = 0.578, 95% CI: 0.464–0.719, *p* < 0.001), chemotherapy (HR = 0.522, 95% CI: 0.380–0.717, *p* < 0.001), and systemic therapy (HR = 0.718, 95% CI: 0.517–0.997, *p* = 0.048) were all confirmed as independent favorable prognostic factors.

**FIGURE 3:**
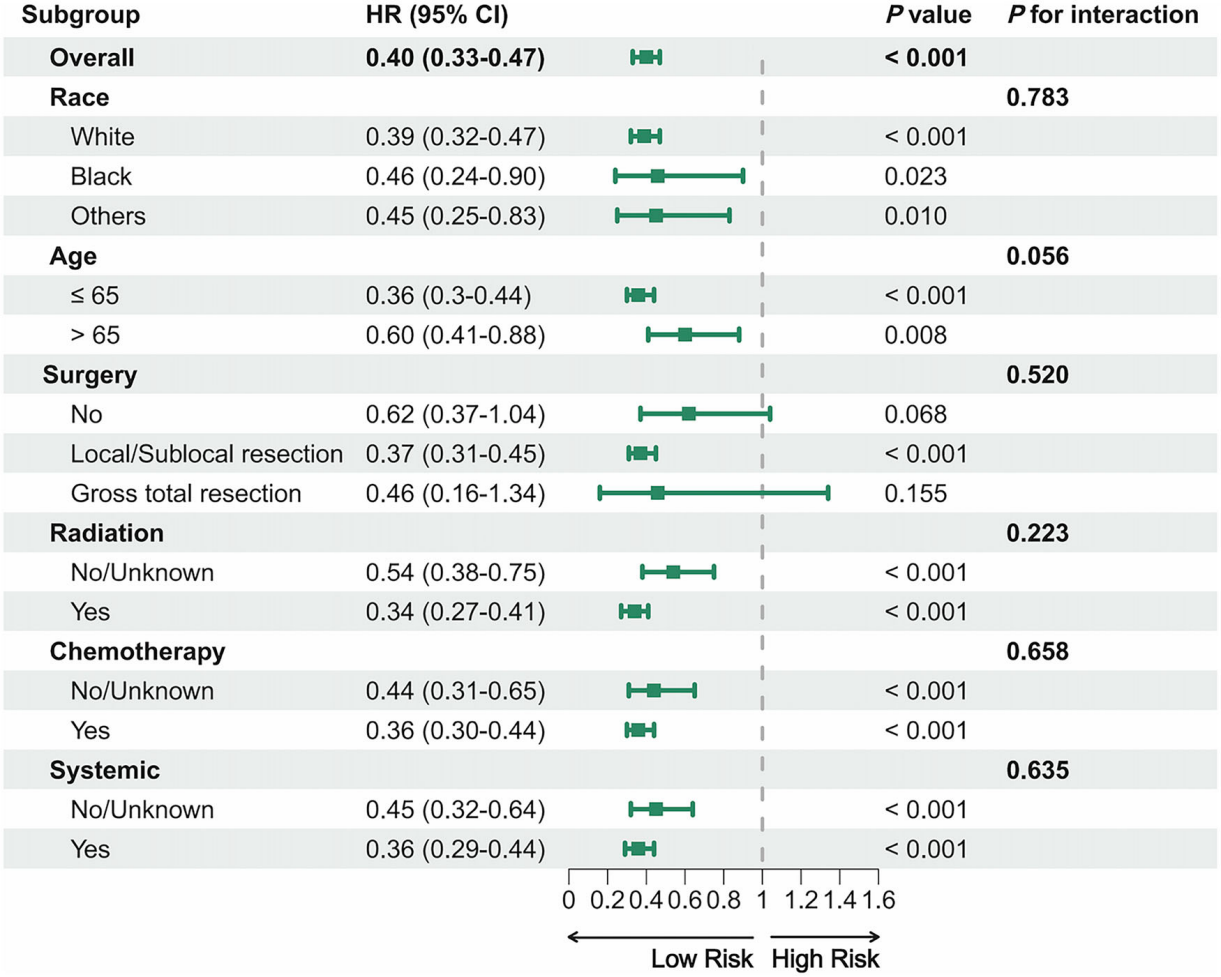
Forest plot of subgroup analysis for overall survival in the PSM‐matched cohort.

Subgroup analysis further corroborated the robustness of these results (**Figure** **3**). The survival advantage conferred by IDH mutation persisted across strata defined by demographic factors (race, age) and treatment factors (chemotherapy, surgery, radiotherapy, systemic therapy). Notably, this benefit remained significant within the chemotherapy subgroup, suggesting that this molecular signature maintains stable prognostic stratification capability in the context of standard‐of‐care treatment. Crucially, interaction analyses revealed that this survival benefit was highly robust and consistent across diverse clinical contexts. The “*p* for interaction” values for all examined demographic and treatment strata were consistently greater than 0.05, demonstrating no significant interactive confounding.

### 3.4 Construction and Performance Evaluation of ML Prognostic Models

#### 3.4.1 Variable Integration and Multi‐Model Screening

**FIGURE 4:**
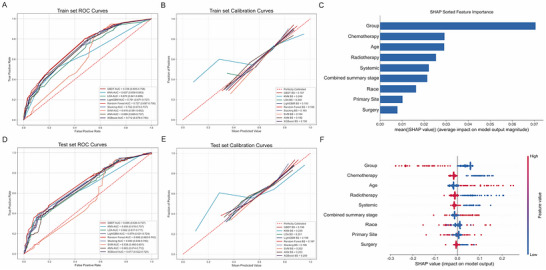
Performance evaluation and interpretability analysis of the RF prognostic model. The ROC curves of 9 ML algorithms in the training set (A) and testing set (D). Calibration curves of the RF model in the training set (B) and testing set (E). (C) Feature importance ranking based on mean absolute SHAP values. (F) SHAP summary plot visualizing the direction and magnitude of each feature's impact on mortality risk; red dots indicate high feature values, and blue dots indicate low feature values.

To construct a precise prognostic stratification tool for GBM patients, nine independent clinical variables identified via the Model 3 were integrated (**Table** **3**). Nine mainstream ML algorithms were trained and compared, with each model undergoing fivefold cross‐validation. Initial evaluation using the AUC revealed that the RF model achieved the highest AUC values in both the training (**Figure** **4A**) and testing (**Figure** **4D**) sets. Considering that AUC primarily assesses discrimination and may not fully reflect clinical utility (Muschelli 2020), Calibration Curves and Decision Curve Analysis (DCA) were further analyzed. The calibration curves showed that the RF model achieved superior accuracy in predicted probabilities across both the training (**Figure** **4B**) and test (**Figure** **4E**) sets. Furthermore, DCA confirmed that the RF model provided the highest net benefit across a wide range of threshold probabilities (**Figure** **S3**). Collectively, these findings established the RF model as the optimal prognostic tool for this study.

**TABLE 3:** Comparative performance evaluation of nine machine learning algorithms in the training and testing sets.

Models	Train					Test				
	AUC	Sensitivity	Specificity	Accuracy	*F*1	AUC	Sensitivity	Specificity	Accuracy	*F*1
GBDT	0.726	0.730	0.705	0.727	0.823	0.694	0.710	0.615	0.699	0.807
KNN	0.637	0.744	0.417	0.604	0.681	0.657	0.741	0.420	0.607	0.687
LDA	0.670	0.723	0.647	0.713	0.814	0.661	0.723	0.630	0.710	0.811
LightGBM	0.701	0.725	0.657	0.716	0.816	0.678	0.720	0.615	0.706	0.808
RF	0.727	0.735	0.717	0.732	0.826	0.698	0.714	0.600	0.699	0.805
Stacking	0.701	0.730	0.682	0.723	0.820	0.689	0.725	0.661	0.716	0.816
SVM	0.615	0.740	0.741	0.740	0.831	0.538	0.728	0.661	0.718	0.816
ANN	0.699	0.729	0.655	0.719	0.816	0.664	0.716	0.582	0.697	0.802
XGBoost	0.712	0.730	0.673	0.722	0.819	0.676	0.718	0.588	0.699	0.803

#### 3.4.2 Detailed Performance Evaluation of the Optimal RF Model

Quantitative assessment of the RF model was performed using fivefold cross‐validation. The results demonstrated robust predictive performance, with mean AUCs of 0.727 (95% CI: 0.697–0.756) in the training set and 0.698 (95% CI: 0.662–0.742) in the test set. To rigorously evaluate model calibration quantitatively, Brier scores were calculated, achieving 0.186 in the training set and 0.197 in the testing set, which indicated a favorable agreement between the predicted probabilities and actual outcomes. Confusion matrices were used to evaluate classification efficacy further. In the training set, the model achieved a correctness of 0.743 and a precision of 0.736 (**Figure** **S4A**). In the testing set, the model maintained a correctness of 0.702 and an accuracy of 0.714 (**Figure** **S4B**). Overall, the proposed model demonstrated efficiency and stability in stratifying patients into high‐ and low‐risk groups.

#### 3.4.3 SHAP‐Based Interpretability Analysis

To elucidate the “black box” of machine learning and visualize the impact of key variables, SHAP (Shapley Additive exPlanations) analysis was employed (Lundberg et al. 2020). The feature importance ranking based on mean absolute SHAP values (**Figure** **4C**) identified group (IDH mutation status), chemotherapy, and age as the top three contributors to the model's predictions. This finding algorithmically corroborates the status of IDH mutation as a primary prognostic driver. Additionally, the SHAP summary plot (**Figure** **4F**) visualized the directionality of these effects: belonging to the IDH‐mutant group and receiving chemotherapy were associated with lower SHAP values (indicating reduced mortality risk), whereas advanced age significantly increased the predicted risk (indicated by red points in the high‐risk region). These findings align perfectly with the results from the conventional Cox regression analysis, reinforcing the scientific validity and interpretability of the machine learning model in a clinical context.

## 4 Discussion

This study leverages high‐fidelity data from the Surveillance, Epidemiology, and End Results (SEER) “Transition Era” (2018–2022) to provide definitive real‐world validation for the clinical rationale behind the 2021 World Health Organization (WHO) Classification of Tumors of the Central Nervous System (Louis et al. 2021; Tesileanu et al. 2021). While the favorable prognostic nature of IDH mutations is biologically well‐established, the incremental scientific contribution of this study lies in bridging the critical gap between highly controlled clinical trials and unselected, real‐world populations. Previous studies, constrained by the broad historical code (9440/3), were frequently confounded by “phenotypic heterogeneity” resulting from the admixture of wild‐type tumors—a limitation recurrently noted in statistical reports from the Central Brain Tumor Registry of the United States (CBTRUS) (Ostrom et al. 2022). By targeting the specific 9445/3 code during this unique coding “transition era” and employing doubly robust statistical inference, we successfully “refined” the study cohort. This approach provides large‐scale, population‐level epidemiological validation that the theoretical survival benefits of the newly implemented 2021 WHO diagnostic framework (specifically, “Astrocytoma, IDH‐mutant, grade 4”) consistently and robustly translate into real‐world prognostic stratification, free from historical phenotypic confounding.

Analysis showed that patients with Isocitrate Dehydrogenase (IDH) mutations had a 65.0% lower risk of mortality (HR = 0.350) compared to the NOS group. This magnitude of benefit, surpassing historical estimates, confirms that segregating IDH‐mutant tumors from “Glioblastoma (GBM)” is not merely a terminological update but a recognition of a biologically distinct entity (Brat et al. 2020; Reuss et al. 2015). The substantial prognostic divergence observed in our epidemiological analysis aligns with the well‐established biological distinctiveness of IDH mutations, which have been shown in prior molecular studies to drive fundamentally different oncogenic trajectories compared to wild‐type tumors (Turcan et al. 2012; Waitkus et al. 2018; Pirozzi and Yan 2021). While registry data cannot elucidate direct molecular mechanisms, our real‐world findings strongly corroborate the clinical manifestation of these inherent biological differences, supporting the concept that genotype carries greater weight than traditional histological phenotype in defining the natural history of these tumors (Reuss et al. 2015).

Given the favorable prognosis of IDH‐mutant high‐grade GBM, determining the optimal intensity of adjuvant therapy remains a critical dilemma in clinical management. Our doubly robust multivariable analysis and SHAP‐based model provide crucial insights into this debate. In our study, chemotherapy was identified as a strongly influential prognostic feature (HR = 0.522), trailing only IDH status. However, this finding warrants cautious interpretation. As an observational study, our data are susceptible to indication bias and residual confounding. Key variables that heavily dictate treatment assignment, such as Karnofsky Performance Status (KPS), MGMT promoter methylation status, and exact extent of resection, are unavailable in the SEER registry. Therefore, the observed strong protective association for chemotherapy likely reflects, at least in part, patient selection bias (i.e., healthier patients with favorable profiles being preferentially selected for aggressive treatment) rather than pure causal efficacy. Consequently, our findings highlight a strong statistical association but should not be definitively interpreted as an argument against therapeutic de‐escalation (Weller et al. 2021), which requires validation in prospective, controlled clinical trials. Therefore, the observed survival advantage associated with chemotherapy likely reflects, at least in part, patient selection bias (i.e., healthier patients with favorable profiles being preferentially selected for aggressive treatment) rather than a definitive causal therapeutic effect. Consequently, our findings highlight a strong prognostic association but should not be definitively interpreted as a causal mandate against therapeutic de‐escalation (Weller et al. 2021), which requires validation in prospective, controlled clinical trials. Nevertheless, this real‐world epidemiological association aligns with long‐term results from the CATNON and NOA‐04 trials (Van Den Bent et al. 2021; Wick et al. 2016), reinforcing that IDH‐mutant cohorts derive substantial survival benefits from adjuvant alkylating agent therapy. Furthermore, advanced age (>65 years) was identified as a potent negative prognostic factor (HR = 2.159), suggesting that elderly patients represent a vulnerable subgroup in which the “IDH protective effect” is attenuated, necessitating careful, individualized clinical management rather than standard aggressive regimens.

Traditional Cox proportional hazards models are predicated on assumptions of linearity and log‐linearity, whereas biological systems frequently exhibit complex nonlinear interactions. By deploying ensemble machine learning algorithms, specifically the Random Forest (RF) model, we transcended these statistical limitations. The RF model not only achieved robust predictive performance (test set AUC = 0.698) but also, through SHAP analysis, provided a transparent window into the hierarchical structure of prognostic factors. While this interpretability analysis largely reiterates known clinical predictors rather than discovering novel biological markers, its incremental value lies in its unbiased, data‐driven nature. Notably, without any prior biomedical knowledge input or explicit programming, the algorithm autonomously identified “Group (IDH status)” as the highest‐weighted predictor—constituting a robust “computational proof‐of‐reliability” and algorithmic validation of the WHO 2021 classification (Appay et al. 2019). This convergence of algorithmic intelligence and neuropathological consensus confirms that our model captures genuine signals of disease progression rather than merely fitting statistical noise, thereby offering an interpretable tool for precise clinical risk stratification, a conclusion similarly corroborated by other recent deep learning prognostic studies (Senders et al. 2020).

The core strength of this study lies in the utilization of the novel SEER 9445/3 code, combined with PSM and IPTW strategies, which significantly enhanced the reliability of statistical inference. However, limitations remain. First, the SEER database lacks granular information on key molecular biomarkers, such as MGMT promoter methylation and CDKN2A deletion, limiting comprehensive molecular‐level analysis (Appay et al. 2019). Second, we must acknowledge the potential for contamination within the control group. As molecular testing was not universally exhaustive, cryptic IDH‐mutant cases may default to the NOS (9440/3) category. To formally quantify the impact of such misclassification, we conducted a formal Monte Carlo sensitivity simulation across various diagnostic sensitivity scenarios (**Table** **S4**). The simulation demonstrated that even if up to 30% of true IDH‐mutant cases were cryptically misclassified into the NOS cohort, the corrected prognostic benefit remains highly robust (Corrected HR = 0.562). Furthermore, to address the potential for unmeasured confounding (such as unavailable KPS or MGMT status), we calculated an *E*‐value for our multivariable model. The resulting *E*‐value was 4.04 (lower bound of the 95% confidence interval = 3.33). This indicates that an unmeasured confounder would need to be associated with both IDH status and mortality by an exceptionally high risk ratio of 4.04—a magnitude rarely observed in clinical oncology—to entirely nullify the observed survival advantage. Together, these quantitative bias analyses confirm that our reported survival benefit (Model 3, HR = 0.350) is highly conservative and robust against both misclassification and residual confounding.

Furthermore, our study is inherently constrained by the coding schema of the SEER registry during the 2018–2022 transition era. Because the ICD‐O‐3 code 9445/3 functioned as the primary proxy for IDH‐mutant grade 4 tumors, we were unable to extract a distinct, parallel cohort explicitly coded as “Astrocytoma, IDH‐mutant, grade 4” for direct comparative analysis. Future investigations utilizing prospective registries with fully updated WHO 2021 nomenclatures are warranted to statistically confirm the clinical equivalence between the historical “IDH‐mutant GBM” and the newly defined “Astrocytoma, IDH‐mutant, grade 4” entities. Finally, we acknowledge that the predictive performance of our Random Forest model is modest (test AUC = 0.698). This performance ceiling is inherently constrained by the limited, low‐dimensional clinical variables available in the SEER registry. More importantly, the absence of an external validation cohort represents a major limitation that restricts the immediate generalizability of the proposed prognostic signature. Consequently, this machine learning model remains strictly exploratory. It cannot be deployed for immediate clinical decision‐making or generalized to broader populations without rigorous external validation in independent, prospective multi‐center cohorts.

In conclusion, this study provides compelling real‐world evidence corroborating the superior clinical value of the WHO 2021 reclassification of IDH‐mutant GBM. IDH mutation status serves as the most potent independent favorable prognostic indicator for this population. Our integrated model reveals a strong association between aggressive multimodal management and maximized survival outcomes, though future prospective trials are required to fully account for patient selection biases. The prognostic tool developed herein holds promise for refining risk stratification and guiding personalized treatment decision‐making in the molecular era of neuro‐oncology.

## Author Contributions

**Amu Jike**: software, formal analysis, investigation. **Dongnan Yu**: formal analysis, software. **Shun Li**: funding acquisition, writing – review and editing, project administration, supervision. **Shizheng Tan**: formal analysis, data curation. **Shuming Wang**: data curation, software. **Dewei Du**: writing – original draft, conceptualization, data curation, formal analysis.

## Funding

The current study was supported by grants from the Sichuan Medical Science and Technology Innovation Research Association (YCH‐KY‐YCZD2024‐054), the Health China BuChang ZhiYuan Public welfare projects for Heart and brain health of Zhongshe Social Work Development Foundation (HIGHER2024056), the Affiliated Hospital of North Sichuan Medical College (2019ZD001), the North Sichuan Medical College (CBY23‐ZDA01), and the Bureau of Science and Technology Nanchong City (19SXHZ0321).

## Ethics Statement

We obtained the data for this study from the publicly available SEER database. Our study complied with all relevant ethical laws and institutional guidelines. All individuals involved provided written consent to participate in this research.

## Conflicts of Interest

The authors declare that the research was conducted in the absence of any commercial or financial relationships that could be construed as a potential conflict of interest.

## Artificial Intelligence Usage Declaration

The authors declare that no artificial intelligence (AI) tools, machine learning technologies, or large language models (LLMs) were used in the preparation, data analysis, drafting, or editing of this manuscript.

## Supporting information




**Table S1**. Detailed configurations of hyperparameter tuning for the nine machine learning algorithms.

**Table S2**. Balance of baseline characteristics in the weighted pseudo‐population after IPTW.

**Table S3**. Identification of independent prognostic factors based on multivariable Cox proportional hazards analysis (Model 3) in the PSM‐matched cohort.

**Table S4**. Quantitative bias analysis and Monte Carlo sensitivity simulation for potential misclassification.

**Figure S1**. Diagnostic plots for the Doubly Robust Cox proportional hazards model (Model 3). The plots display the dfbeta residuals for all 14 covariates, demonstrating the absence of extreme influential observations that could disproportionately drive the hazard ratios.

**Figure S2**. Assessment of covariate balance. Love plot showing SMD of baseline characteristics before and after PSM.

**Figure S3**. The DCA of the prognostic model. (A) Training set and (B) testing set.

**Figure S4**. Confusion matrix classification performance of the RF model.

## Data Availability

The raw data supporting the conclusions of this article will be made available by the authors, without undue reservation.
