# Supplementary material for: Real‐World Validation of the Clinical Distinctiveness of IDH‐Mutant Glioblastoma in the SEER Transition Era: A Population‐Based Study Integrating Machine Learning
Source: Brain Behav. 2026 Aug 11;16(8):e71641. doi: 10.1002/brb3.71641 (PMC13462303; doi:10.1002/brb3.71641)
Supplement: Supplementary file 1 [file BRB3-16-e71641-s001.docx]

| Characteristics |  | Multivariate analysis | | |
| --- | --- | --- | --- | --- |
|  | Number(%) | HR | (95%CI) | *P*-value |
| Race |  |  |  |  |
| White | 1289 (83.1%) | Ref |  |  |
| Black | 122 ( 7.9%) | 1.060 | 0.837-1.344 | 0.627 |
| Others | 141 ( 9.0%) | 0.751 | 0.594-0.949 | **0.017** |
| Age |  |  |  |  |
| ≤ 65 | 1301 (83.8%) | Ref |  |  |
| > 65 | 251 (16.2%) | 2.159 | 1.828-2.551 | **< 0.001** |
| Marital status |  |  |  |  |
| Single | 474 (30.5%) | Ref |  |  |
| Married | 924 (59.5%) | 0.922 | 0.796-1.068 | 0.279 |
| Others | 154 ( 10.0%) | 0.952 | 0.760-1.193 | 0.670 |
| Sex |  |  |  |  |
| Female | 581 (37.4%) | Ref |  |  |
| Male | 971 (62.6%) | 1.033 | 0.904-1.180 | 0.633 |
| Median household income |  |  |  |  |
| < 100000 | 1047 (67.5%) | Ref |  |  |
| ≥ 100000 | 505 (32.5%) | 1.023 | 0.894-1.171 | 0.736 |
| Primary site |  |  |  |  |
| Supratentorial lobes | 1324 (85.3%) | Ref |  |  |
| Cerebellum and brain stem | 11 ( 0.7%) | 1.205 | 0.527-2.753 | 0.685 |
| Ventricle | 6 ( 0.4%) | 3.069 | 1.083-8.696 | **0.035** |
| Overlapping lesion of brain | 154 ( 9.9%) | 0.899 | 0.658-1.227 | 0.502 |
| Brain, NOS | 57 ( 3.7%) | 1.019 | 0.653-1.591 | 0.933 |
| Laterality |  |  |  |  |
| Left | 670 (43.2%) | Ref |  |  |
| Right | 675 (43.5%) | 1.003 | 0.877-1.146 | 0.969 |
| Others | 207 (13.3%) | 1.298 | 0.929-1.813 | 0.127 |
| Combined summary stage |  |  |  |  |
| Localized | 1254 (80.8%) | Ref |  |  |
| Regional | 260 (16.8%) | 1.425 | 1.212-1.676 | **< 0.001** |
| Distant | 11 ( 0.7%) | 1.973 | 1.000-3.894 | **0.050** |
| Others | 27 ( 1.7%) | 1.015 | 0.621-1.662 | 0.951 |
| Tumor size(cm) |  |  |  |  |
| < 4.5 | 521 (33.5%) | Ref |  |  |
| ≥ 4.5 | 788 (50.8%) | 1.032 | 0.896-1.190 | 0.659 |
| Unknown | 243 (15.7%) | 0.808 | 0.664-0.984 | 0.064 |
| Surgery |  |  |  |  |
| No | 112 ( 7.2%) | Ref |  |  |
| Local/Sublocal resection | 1395 (89.9%) | 0.637 | 0.459-0.883 | **0.007** |
| Gross total resection | 45 ( 2.9%) | 0.594 | 0.368-0.959 | **0.033** |
| Radiotherapy |  |  |  |  |
| No/Unknown | 279 (18.0%) | Ref |  |  |
| Yes | 1273 (82.0%) | 0.578 | 0.464-0.719 | **< 0.001** |
| Chemotherapy |  |  |  |  |
| No/Unknown | 225 (14.5%) | Ref |  |  |
| Yes | 1327 (85.5%) | 0.522 | 0.380-0.717 | **< 0.001** |
| Systemic |  |  |  |  |
| No/Unknown | 255 (16.4%) | Ref |  |  |
| Yes | 1297 (83.6%) | 0.718 | 0.517-0.997 | **0.048** |
| Group |  |  |  |  |
| GBM NOS | 1240 (79.9%) | Ref |  |  |
| GBM IDH-mutant | 312 (20.1%) | 0.350 | 0.293-0.418 | **< 0.001** |
