# Supplementary material for: Real‐World Validation of the Clinical Distinctiveness of IDH‐Mutant Glioblastoma in the SEER Transition Era: A Population‐Based Study Integrating Machine Learning
Source: Brain Behav. 2026 Aug 11;16(8):e71641. doi: 10.1002/brb3.71641 (PMC13462303; doi:10.1002/brb3.71641)
Supplement: Supplementary file 2 [file BRB3-16-e71641-s005.docx]

| Machine Learning Algorithm | Hyperparameter | Search Grid (Range) | Optimal Setting |
| --- | --- | --- | --- |
| ANN | hidden_layer_sizes | (2, 2), (4, 4), (8, 8), (10, 10) | **(4, 4)** |
|  | max_iter | 500, 1000, 1500 | **1000** |
| GBDT | n_estimators | 50, 60, ..., 200 | **140** |
|  | learning_rate | 0.001, 0.01, 0.1, 1.0 | **0.01** |
|  | max_depth | 3, 4, 5, 6, 7 | **7** |
|  | min_samples_split | 2, 3, ..., 10 | **8** |
|  | min_samples_leaf | 1, 2, 3, 4, 5 | **3** |
| KNN | n_neighbors | 1, 2, ..., 10 | **7** |
|  | weights | "uniform", "distance" | **"uniform"** |
|  | p | 1, 2 | **1** |
| LDA | solver | "svd", "lsqr", "eigen" | **"lsqr"** |
|  | shrinkage | None, "auto", 0.1, 0.5, 0.9 | **0.1** |
| LGBM | learning_rate | 0.01, 0.05, 0.1 | **0.01** |
|  | num_leaves | 31, 40, 50 | **50** |
|  | max_depth | -1, 5, 10 | **10** |
|  | n_estimators | 100, 200, 300 | **300** |
|  | subsample | 0.8, 0.9, 1.0 | **0.8** |
|  | colsample_bytree | 0.8, 0.9, 1.0 | **0.9** |
|  | reg_alpha | 0, 0.1, 0.5 | **0.5** |
|  | reg_lambda | 0, 0.1, 0.5 | **0.1** |
| RF | n_estimators | 10, 20, ..., 200 | **190** |
|  | max_depth | None, 3, 4, ..., 13 | **9** |
|  | min_samples_split | 2, 3, ..., 10 | **2** |
|  | min_samples_leaf | 1, 2, 3, 4, 5 | **2** |
|  | max_features | "auto", "sqrt", "log2", None, 1, 2, ..., 14 | **3** |
| Stacking | decision_tree__max_depth | 3, 5, 7 | **7** |
|  | svm__C | 0.1, 1, 10 | **0.1** |
|  | svm__kernel | "linear", "rbf" | **"rbf"** |
|  | final_estimator__C | 0.1, 1, 10 | **10** |
| SVM | C | 0.001, 0.01, 0.1, 1, 10, 100 | **10** |
|  | gamma | 0.001, 0.01, 0.1, 1, 10, 100 | **10** |
|  | kernel | "linear", "rbf", "sigmoid" | **"rbf"** |
| XGBoost | learning_rate | 0.01, 0.05, 0.1 | **0.05** |
|  | max_depth | 3, 6, 9 | **3** |
|  | n_estimators | 50, 100, 200 | **100** |
|  | subsample | 0.8, 0.9, 1.0 | **1.0** |
|  | colsample_bytree | 0.8, 0.9, 1.0 | **0.9** |
