# Supplementary material for: Real‐World Validation of the Clinical Distinctiveness of IDH‐Mutant Glioblastoma in the SEER Transition Era: A Population‐Based Study Integrating Machine Learning
Source: Brain Behav. 2026 Aug 11;16(8):e71641. doi: 10.1002/brb3.71641 (PMC13462303; doi:10.1002/brb3.71641)
Supplement: Supplementary file 3 [file BRB3-16-e71641-s007.docx]

| Characteristic | After IPWT | | *P*-value | SMD |
| --- | --- | --- | --- | --- |
|  | GBM NOS  N = 13442.9 | GBM IDH-mutant  N = 13821.2 |  |  |
| Race |  |  | 0.885 | 0.041 |
| White | 11729.9 (87.3%) | 11937.5 (86.4%) |  |  |
| Black | 814.0 (6.1%) | 976.9 (7.0%) |  |  |
| Others | 899.0 (6.6%) | 906.7 (6.6%) |  |  |
| Age |  |  | 0.591 | 0.048 |
| ≤ 65 | 6901.8 (51.3%) | 6765.1 (48.9%) |  |  |
| > 65 | 6541.1 (48.7%) | 7056.1 (51.1%) |  |  |
| Marital status |  |  | 0.666 | 0.074 |
| Single | 2289.0 (17.0%) | 2140.1 (15.5%) |  |  |
| Married | 8910.9 (66.3%) | 9013.3 (65.2%) |  |  |
| Others | 2243.0 (16.7%) | 2667.8 (19.3%) |  |  |
| Sex |  |  | 0.310 | 0.091 |
| Female | 5558.6 (41.4%) | 5102.5 (36.9%) |  |  |
| Male | 7884.2 (58.6%) | 8718.7 (63.1%) |  |  |
| Median household income($) |  |  | 0.999 | < 0.001 |
| < 100000 | 9233.7 (68.7%) | 9494.6 (68.7%) |  |  |
| ≥ 100000 | 4209.2 (31.3%) | 4326.6 (31.3%) |  |  |
| Primary site |  |  | 0.084 | 0.226 |
| Supratentorial lobes | 10847.6 (80.7%) | 10701.8 (77.4%) |  |  |
| Cerebellum and brain stem | 139.0 (1.1%) | 129.4 (0.9%) |  |  |
| Ventricle | 47.0 (0.3%) | 34.8 (0.3%) |  |  |
| Overlapping lesion of brain | 1546.3 (11.5%) | 2496.4 (18.1%) |  |  |
| Brain, NOS | 863.0 (6.4%) | 458.6 (3.3%) |  |  |
| Laterality |  |  | 0.633 | 0.089 |
| Left | 5404.1 (40.2%) | 5631.3 (40.7%) |  |  |
| Right | 5640.6 (42.0%) | 5307.4 (38.4%) |  |  |
| Others | 2398.2 (17.8%) | 2882.5 (20.9%) |  |  |
| Combined summary stage |  |  | 0.498 | 0.146 |
| Localized | 10916.6 (81.2%) | 10710.2 (77.5%) |  |  |
| Regional | 2124.2 (15.8%) | 2332.5 (16.9%) |  |  |
| Distant | 145.0 (1.1%) | 384.1 (2.7%) |  |  |
| Others | 257.1 (1.9%) | 394.4 (2.9%) |  |  |
| Tumor size(cm) |  |  | 0.769 | 0.060 |
| < 4.5 | 6037.0 (44.9%) | 5804.7 (42.0%) |  |  |
| ≥ 4.5 | 5809.7 (43.2%) | 6241.2 (45.2%) |  |  |
| Unknown | 1596.2 (11.9%) | 1775.3 (12.8%) |  |  |
| Surgery |  |  | 0.762 | 0.068 |
| No | 2108.1 (15.7%) | 2482.1 (18.0%) |  |  |
| Local/Sublocal resection | 11031.8 (82.1%) | 10973.3 (79.4%) |  |  |
| Gross total resection | 303.0 (2.2%) | 365.8 (2.6%) |  |  |
| Radiotherapy |  |  | 0.274 | 0.092 |
| No/Unknown | 2943.9 (21.9%) | 2519.1 (18.2%) |  |  |
| Yes | 10499.0 (78.1%) | 11302.1 (81.8%) |  |  |
| Chemotherapy |  |  | 0.350 | 0.087 |
| No/Unknown | 3397.1 (25.3%) | 2983.8 (21.6%) |  |  |
| Yes | 10045.8 (74.7%) | 10837.4 (78.4%) |  |  |
| Systemic |  |  | 0.937 | 0.008 |
| No/Unknown | 4473.1 (33.3%) | 4546.4 (32.9%) |  |  |
| Yes | 8969.8 (66.7%) | 9274.8 (67.1%) |  |  |
