# Supplementary material for: Real‐World Validation of the Clinical Distinctiveness of IDH‐Mutant Glioblastoma in the SEER Transition Era: A Population‐Based Study Integrating Machine Learning
Source: Brain Behav. 2026 Aug 11;16(8):e71641. doi: 10.1002/brb3.71641 (PMC13462303; doi:10.1002/brb3.71641)
Supplement: Supplementary file 4 [file BRB3-16-e71641-s008.docx]

| Simulation Scenario (Diagnostic Sensitivity) | Simulated Cryptic IDH-Mutant Misclassification Rate | HR | Shift Direction |
| --- | --- | --- | --- |
| Baseline (Uncorrected) | Baseline (Uncorrected) | 0.434 | N/A |
| Sensitivity = 0.95 | 5% | 0.452 | Toward Null (1.0) |
| Sensitivity = 0.90 | 10% | 0.473 | Toward Null (1.0) |
| Sensitivity = 0.85 | 15% | 0.494 | Toward Null (1.0) |
| Sensitivity = 0.80 | 20% | 0.512 | Toward Null (1.0) |
| Sensitivity = 0.70 | 30% | 0.562 | Toward Null (1.0) |

*(Note below the table)* **Note:** A Monte Carlo simulation (20 iterations per scenario) was performed to model the impact of undetected IDH-mutant cases cryptically defaulting to the NOS category. The results quantitatively demonstrate that even under severe misclassification scenarios (e.g., 30% missed mutations), the IDH mutation remains a robust, favorable prognostic factor. Additionally, the calculated **E-value of 4.04** (lower bound of confidence interval: 3.33) further demonstrates robust resistance to potential unmeasured confounding.
